# Effect of Gelled Emulsions Elaborated with Soybean Oil, Maca (*Lepidium meyenni*) Flour, and Chincho (*Tagetes elliptica* Sm.) Essential Oil upon Animal Fat Substitution in Beef Burgers

**DOI:** 10.3390/foods11152198

**Published:** 2022-07-24

**Authors:** Francis Cerrón-Mercado, Carmen M. Botella-Martínez, Bettit K. Salvá-Ruíz, Juana Fernández-López, Jose A. Pérez-Alvarez, Manuel Viuda-Martos

**Affiliations:** 1 IPOA Research Group, Agro-Food Technology Department, Centro de Investigación e Innovación Agroalimentaria y Agroambiental (CIAGRO-UMH), Miguel Hernández University, Orihuela, 03312 Alicante, Spain; francis.cerron@goumh.umh.es (F.C.-M.); c.botella@umh.es (C.M.B.-M.); j.fernandez@umh.es (J.F.-L.); ja.perez@umh.es (J.A.P.-A.); 2 Departamento de Tecnología de Alimentos y Productos Agropecuarios (TAPA), Universidad Nacional Agraria la Molina, UNALM, Lima 15024, Peru; bsalva@lamolina.edu.pe

**Keywords:** healthy burgers, gelled emulsion, chincho (*Tagetes elliptica* Sm.) essential oil, soja oil, fat replacement, fatty acid profile, maca (*Lepidium meyenni*) flour

## Abstract

The aim of this study was to analyze the effect of pork backfat (PB) substitution in a meat burger with a gelled emulsion (GE) elaborated with maca flour, soybean oil, and chincho essential oil (CEO). Lipid profile (gas chromatography—GC), health indices, physicochemical properties (CIELAB color, pH, texture profile—TPA), and cooking and sensory characteristics of meat burgers were analyzed. Five formulations were evaluated: control (BC) (80% beef meat and 20% PB); BSM (10% PB + 10% GE); BSMC0.25 (BSM + 0.25% CEO); BSMC0.5 (BSM + 0.5% CEO), and BSMC1.0 (BSM + 1.0% CEO). GE substitution in meat burgers provided a healthier lipid profile; the amount of SFA was reduced (*p* < 0.05), while PUFA content was significantly increased (*p* < 0.05). Furthermore, the use of GE resulted in healthier PUFA/SFA ratios and lower atherogenic and thrombogenic indices. The addition of GE increased moisture content and decreased fat and protein contents. Color parameters (L*, b*, and C*) decreased after cooking. Hardness (*p* < 0.05), cooking losses, and shrinkage changes decreased with GE addition. Lipid oxidation levels were significantly (*p* < 0.05) affected by GE substitution. Therefore, the substitution of PB by GE can be considered as an effective strategy to produce healthier meat burgers without negatively affecting their physicochemical and technological properties.

## 1. Introduction

Nowadays, meat burgers are one of the most popular products; however, their consumption in excess is related to unhealthy habits due to their high content of saturated fatty acids (SFA). These fatty acids are related to the risk of chronic, carcinogenic, and degenerative diseases [1,2,3]. COVID-19 provoked an increase in interest in eating healthier foods and meat products were not the exception. Several studies have shown that consumers are willing to consume new or reformulated healthy meat products [4,5]. A feasible alternative to this situation is to replace fatty tissues (belly, lard, etc.) with gelled emulsions (GE). These emulsions are made with polyunsaturated oils such as walnut, almond, chia, hemp, soybean, sunflower, wheat, or algae oils, which are healthier than animal fats. The development of GE generated by gelation procedures can ensure health-enhancing nutritional properties and could reduce cardiovascular diseases within a balanced diet [6,7], without the loss of technological characteristics and sensory properties, making these types of products well appreciated by the consumers [5,8]. Some authors have obtained promising results for GE using various vegetable oils with healthy lipid profiles, such as the previously aforementioned oils with gelling agents such as starch corn, makgeolli, basil gum, gelatin, date flour, and amaranth flour [9,10,11,12,13,14]. All of these have been successfully used in low-fat meat products.

In the development of healthy meat products, gelled emulsions are used as fat analogs. One of the most important aspects when GEs are used is to improve the lipid profile, but their use can change the sensory characteristics and the technological qualities of these type of products [3]. Therefore, it is very important to reformulate this type of product without any loss of important characteristics for consumers and industries [8,9,11]. Animal fat substitution and the development of new healthy meat products presents a healthy and sustainable alternative diet based on traditional meat burgers. Thus, the substitution of PB with soybean oil (*Glycine max*) and chincho (*Tagetes elliptica* Sm.) essential oil could be an attractive, nutritious, and ethical alternative to conventional meat burgers.

Maca (*Lepidium meyenni*) flour has beneficial health effects due to its content of bioactive compounds, including glucosinolates and flavonoids [15]. From a technological point of view, the starch content in maca as a product of fractionation processing could be used as an emulsifier and stabilizer to give foods the desired texture and consistency [16]. Furthermore, antioxidants derived from maca could be used to prevent lipid-rich foods from developing rancidity and to control enzymatic browning of fresh produce [17]. Soybean oil is a worldwide and well-known oil for its content of tocopherols and polyunsaturated fatty acids, among other bioactive compounds [18]. The most important polyunsaturated fatty acids found in soybean oil are linolenic and linoleic acids, while oleic acid is the main monounsaturated fatty acid [19]. Thus, due to this composition, soybean oil could be a good lipid source for the elaboration of gelled emulsions to be used as fat replacers. On the other hand, healthy meat product developers must take into account that GEs elaborated with polyunsaturated oils are susceptible to lipid oxidation with unpleasant meat product characteristics such as rancidity, off flavors, and discolorations, among others [20]. To avoid these negative aspects, essential oils could be an excellent alternative to avoid lipid oxidation in healthy meat product development [21]. Several studies have shown that essential oils obtained from plants of the *Tagetes* genus have demonstrated antioxidant and antimicrobial properties [22,23,24]; for this reason, the use of the essential oil of *Tagetes elliptica* Sm. could be a good option in the formulation of healthy meat burgers rich in polyunsaturated fatty acids. *T. elliptica*, the binomial name of Chincho, is an ethnic aromatic plant cultivated in several regions of Central and South America [25]. It has been used for many years as a species to enhance flavor in meat seasoning [26]. Thus, the essential oil obtained from chincho could give healthy meat burgers antioxidant and antimicrobial properties and aromatic compounds [27]. In this way, the elaboration of meat burgers partially substituted with GEs elaborated with soybean oil, maca flour, and chincho essential oil could be an excellent natural vehicle to improve the lipid profile of meat products, and represent a promising alternative to the gelled emulsions currently used in emulsion-type applications.

The aim of this study was to analyze the effect of partially replacing pork backfat with gelled emulsions elaborated with maca flour, soybean oil, and chincho essential oil on chemical composition, physicochemical and cooking properties, and lipid oxidation, as well as the sensory analysis of beef burgers.

## 2. Materials and Methods

### 2.1. Food Materials

In the present study, different gelled emulsions were prepared with the following ingredients: organic Peruvian maca flour (MF) (protein 11.9%, carbohydrates 61.5%, fat 0.7%, and dietary fiber 15.1%) and soybean oil (SO) (48.22% linoleic acid, 30.26% oleic acid, 11.07% palmitic acid, and 5.36% linolenic acid) were purchased in a local supermarket (Orihuela, Spain). Beef meat (72.30% moisture, 1.85% fat, 24.96% protein, and 0.87% ash) and pork backfat (11.20% moisture, 75.60% lipids, 12.43% protein, and 0.77% ash) were acquired from a local butchery provider (Orihuela, Spain). Chincho essential oil was obtained by directed steam distillation of chincho leaves collected in the province of Chupaca, Junin Region, Peru (3263 m above sea level). Gelatin of animal origin (pork) with 180 bloom was obtained from Sosa Ingredients S.L. (Barcelona, Spain)

### 2.2. Preparation of Oil in Water Gelled Emulsions GEs

The gelled emulsions were prepared with maca flour, soybean oil, and chincho essential oil according to Botella-Martinez et al. [28]. Four gelled emulsion were formulated (GE_1_, GE_2_, GE_3_, and GE_4_) and their composition is described in Table 1. The emulsions obtained were kept at 4 °C until the production of the burgers.

### 2.3. Formulation and Processing of Burgers Containing Gelled Emulsions GEs

Five batches of meat burgers were prepared by partially replacing animal fat with gelled emulsions prepared with soybean oil, maca flour and chincho essential oil. A total of 90 burgers (18 burgers for each treatment) with an approximate weight of 29.5 ± 0.05 g each were prepared. The traditional formula was used as a control sample (BC), while for the other four treatments, pork backfat was replaced by a gelled emulsion (GE_1_, GE_2_, GE_3_ and GE_4_), as indicated in Table 2. The samples were shaped with industrial-type burger equipment to obtain samples approximately 0.90 ± 0.05 cm thick and 6.3 ± 0.29 cm in diameter. The burgers were packed into bags and stored at 4 °C until further analysis. Six burgers of each formulation were cooked on a griddle to an internal temperature of 71 °C, taken in the geometrical center of each burger through a hypodermic-type thermometer (Model HVP-2-21-V2-TG-48-OCT-M Omega, Stanford, CT, USA) approximately 2.5 min per side.

### 2.4. Proximate Composition

Moisture, protein (using N × 6.25 as conversion factor), fat, and ash contents were determined according to the official methods of the Association of Official Agricultural Chemists (AOAC) [29].

### 2.5. Lipid Profile and Health Indices

#### 2.5.1. Fatty Acid Profile

To analyze the fatty acids profile, burger fat was obtained from 5 g of sample (raw and cooked burger) according to the methodology of Folch et al. [30]; then, the lipid phase was transmethylated following the method and conditions described by Golay and Moulin [31]. The fatty acid methyl esters (FAMEs) were separated and quantified using a gas chromatograph—Hewlett-Packard 6890—with a flame ionization detector (FID) and a Suprewax 280 capillary column (30 m, 0.25 µm film thickness, 0.25 mm i.d.; Tecknokroma Barcelona, Spain), was carried out according to the chromatographic conditions described by Pellegrini et al. [32], and was expressed as g/100 g of fat.

#### 2.5.2. Health Indices

To evaluate the nutritional quality of burgers, the health indices of beef burgers were calculated. Total fat content and fat composition, measured as total saturated (SFA), monounsaturated (MUFA), and polyunsaturated (PUFA) fatty acids contents, and the n-3 and n-6 fatty acid ratio, the PUFA and SFA ratio were obtained. In the same way, n-6/n-3 and PUFA/SFA ratios and atherogenic index (*AI*), thrombogenic index (*TI*), and hypocholesterolemic/hypercholesterolemic (h/H) were calculated following Equations (1)–(3), respectively, using the equations developed by Ulbricht and Southgate [33].

(1) $$AI=\frac{C12:0+\left(4\times C14:0\right)+C16:0}{\Sigma \;\mathrm{MUFA}+\Sigma \;n-6+\Sigma \;n-3\;}$$

(2) $$TI=\frac{C14:0+C16:0+C18:0}{\left(0.5\times \;\Sigma \;\mathrm{MUFA}\right)+\left(0.5\times \Sigma \;n-6\right)+\left(3\times \;\Sigma \;n-3\right)+\left(\frac{\Sigma \;n-3}{\Sigma \;n-6\;}\right)}$$

(3) $$\frac{h}{H}=\frac{C18:1\;n-9+C18:1\;n-7+\Sigma \;\mathrm{PUFA}}{C14:0+C16:0}$$

### 2.6. Physicochemical Analysis

#### 2.6.1. Color Parameters, pH, and Water Activity

The color of raw and cooked patties was evaluated using CIELAB color space (D_65_ as illuminant and 10° as standard observer) and L*a* b* color coordinates (L*, a*, and b* represent lightness, red/green color, and yellow/blue color, respectively). Samples were measured using a Minolta CM-700 (Minolta Camera Co., Osaka, Japan) using SCI mode and a low-reflectance glass placed on the surface of the sample and equipment. AMSA guidelines for color evaluation were applied [34,35]. Before the measurements, the equipment was calibrated following the equipment recommendations (calibrate plate values of L* = 97.14, a* = 0.14 and b* = 2.40). Six random points from each sample were taken for color determination. The psychophysical magnitudes hue (H*) and chroma (C*) in raw and cooked burgers were also calculated using Equations (4) and (5), respectively.

(4) $$C*=\sqrt{{a*}^{2}+{b*}^{2}}$$

(5) $$H*=arctang\left(\frac{b*}{a*}\right)$$

The total color differences (ΔE*) of each reformulated sample with respect to the control burger were calculated with Equation (6).

(6) $$\Delta E*=\sqrt{{\left(L_{s}*-L_{c}*\right)}^{2}+{\left(a_{s}*-a_{c}*\right)}^{2}+{\left(b_{s}*-b_{c}*\right)}^{2}}$$

where s: sample, and c: control beef burger.

Equations (4)–(6) were obtained according Cassens et al. [36].

Water activity was determined in raw burgers using an electrolytic hygrometer (Novasina TH-500, Novasina, Pfaeffikon, Switzerland) at 22° C. The pH of the samples was measured with a digital portable pH meter using a penetration probe at different sites of the raw and cooked burgers using a Crison model 510 pH meter, (Barcelona, Spain).

#### 2.6.2. Texture Profile Analysis

Texture profile analysis (TPA) was performed in six replicates in cooked burgers. The tests were performed in a TA-XT2i texture analyzer (Stable Micro Systems, Surrey, England). Cubic samples of (2 × 2 × 2 cm) were obtained for fresh and cooked samples, respectively. Samples were compressed to 75% of their original height with a cylindrical probe of 10 cm diameter at a compression load of 25 kg with a constant velocity of 1 mm/s at 15–20 °C. The following parameters were calculated: hardness (N), springiness, cohesiveness, chewiness (N), and gumminess [37].

### 2.7. Cooking Properties

Cooking properties were determined using three burger samples for each treatment. Meat burgers from each batch at room temperature were weighed and their diameters were measured; these procedures were repeated after cooking. The reduction in diameter and the increases in thickness and cooking loss were calculated according to Equations (7)–(9).

(7) $$\mathrm{Shrinkage}\;\left(\%\right)=\frac{\left(\mathrm{raw}\;\mathrm{diameter}-\mathrm{cooked}\;\mathrm{diameter}\right)}{\left(\mathrm{raw}\;\mathrm{diamater}\right)}\;\times 100$$

(8) $$\mathrm{Thickness}\;\mathrm{increase}\;\left(\%\right)=\frac{\left(\mathrm{Cooked}\;\mathrm{thickness}-\mathrm{raw}\;\mathrm{thickness}\right)}{\left(\mathrm{cooked}\;\mathrm{thickness}\right)}\;\times 100$$

(9) $$\left(\%\right)\mathrm{Cooking}\;\mathrm{loss}=\frac{\left(\mathrm{raw}\;\mathrm{weight}-\mathrm{cooked}\;\mathrm{weight}\right)}{\left(\mathrm{raw}\;\mathrm{weight}\right)}\;\times 100$$

### 2.8. Oxidative Stability

The evaluation of lipid stability was performed on raw and cooked burgers by measuring thiobarbituric acid reactive substances (TBARS) following the method proposed by Rosmini et al. [38]. The TBARS value was calculated from a malonaldehyde standard curve expressed as mg of malondialdehyde (MDA)/kg of sample.

### 2.9. Statistical Analysis

Experimental data were expressed as mean ± standard deviation of three repeated measurements per sample (five treatments). Statistical analysis for chemical composition and physicochemical and cooking properties was performed by one-way analysis of variance (ANOVA). Oxidative stability was analyzed by means of a two-way ANOVA test with two factors: thermal treatment (two levels: raw or cooked) and treatments (five levels: BC, BSM, BSMC0.25, BSMC0.5, and BSMC1.0). Tukey’s post hoc test was applied for comparisons of means; statistical significance was accepted at a level of (*p* < 0.05) in all statistical analyses using the software SPSS^®^ IBM^®^ Statistics 22.0.0.0. (International Business Machines Corp., Armonk, New York, NY, USA).

## 3. Results and Discussion

### 3.1. Proximate Composition of Burgers

Table 3 shows the results of the chemical composition of BC and the substitution of 50% of pork fat by the gelled emulsions in BSM, BSMC0.25, BSMC0.5, and BSMC1.0. The moisture content increased in the raw and cooked burgers substituted with the gelled emulsion; the control sample presented significant differences with the reformulations (*p* < 0.05). The increased values of moisture content could be due to the water used to elaborated the gelled emulsions. These results were in agreement with those reported by Lucas-Gonzalez et al. [39] and Botella-Martinez et al. [9] when gelled emulsions are employed in the substitution of fat in meat products.

In reference to the protein content of raw samples (Table 3), the BC showed the highest (*p* < 0.05), while no differences (*p* > 0.05) were obtained between samples where the gelled emulsions were used as fat replacer. The same trend was observed in cooked samples, where BC had the highest (*p* < 0.05) protein values. This reduction in protein content may be due to the fact that in the gelled emulsion, the protein content comes from maca (11.9%). Thus, for every 100 g of emulsion, only 1.7 g of protein is provided, while the pork backfat provides 12.19 g of protein per 100 g. Regarding ash content in raw burgers, no statistical differences (*p* > 0.05) were found between BC and samples with pork backfat partially replaced by gelled emulsions. The cooked burgers of BSM0.25, BSMC0.5, and BSMC1.0 had lower (*p* < 0.05) ash content than BC and BSM, without statistical differences between them (*p* > 0.05). Among the different samples tested, BC showed a higher fat content compared to the burgers partially substituted with the gelled emulsion—BSM, BSMC0.25, BSMC0.5, and BSMC1.0—leading to a decrease in fat content by 41.59% and 7.09% for the BSM sample versus the BC control in the raw and cooked burgers, respectively. This decrease in fat content was similar to that reported in the scientific literature analyzing the substitution of animal fat with a gelled emulsion [9,11,40,41,42].

### 3.2. Lipid Profile and Health Indices

#### 3.2.1. Fatty Acid Profile

The fatty acid profile of raw and cooked beef burgers is shown in Table 4 and Table 5, respectively. In raw burgers, the main saturated fatty acids found in all samples analyzed were palmitic acid (16:0) and stearic acid (18:0). However, significant differences (*p* < 0.05) were obtained between BC, which had the highest values for these fatty acids, and samples where the pork backfat was replace by gelled emulsions. Thus, for palmitic acid, a reduction ranged between 14.07 and 16.84% was obtained with respect to BC, while for stearic acid, the reduction with regards to BC varied between 16.90 and 20.05%. The reduction in saturated fatty acid when gelled emulsions elaborated with healthier oils are used as a fat replacer in meat product is well described in the literature [9,28,41].

Soybean oil has a high content of polyunsaturated fatty acids (PUFA) in its composition (49.24%), followed by monounsaturated fatty acids MUFA (29. 44%). These contents, when incorporated in the gelled emulsion, modify the composition of beef burgers. It was found that the highest MUFA contents in raw and cooked burgers were palmitoleic acid (C16:1) and oleic acid (C18:1), but these were significantly reduced (*p* < 0.05) with the partial substitution of the gelled emulsion in all formulations compared to the control. In this sense, regarding monounsaturated fatty acids, mainly oleic acid (C18:1 (n-9)), the substitution of animal fat with a gelled emulsion elaborated with soybean oil produces a reduction (*p* < 0.05) in the content of this fatty acid with respect to BC.

The most abundant PUFA in raw burgers was linoleic acid (C18:2 n-6); partial substitution of pork backfat (50%) with the gelled emulsion increased the linoleic acid content (*p* < 0.05) in all formulations. In addition, all formulations had significantly higher α-linolenic acid (C18:3 n-3) content (*p* < 0.05) compared to the control in both raw and cooked burgers. These results are in agreement with those reported by Selani et al. [43], who analyzed the effects of pineapple by-products and canola oil used as fat replacers on fatty acid profile. They found that in the samples where the back fat was replaced by the canola oil emulsion or canola oil and pineapple by-products, the fatty acid profile was improved (higher content of MUFA and PUFA and lower content of SFA) with respect to the control sample. Similarly, Szpicer et al. [44] mentioned that in beef burgers where the tallow was partially replaced by canola oil or a mix of canola oil and β-glucan, the oleic, linoleic, and linolenic acids content increased with respect to control samples.

For the cooked samples (Table 5), the trend is very similar to raw samples. Some minor differences in the values, and therefore in the statistical significance in cooked samples with respect to the uncooked samples, may be attributed to the loss of fat and water during cooking.

Burgers with pork backfat (50%) replaced by gelled emulsion (BSM, BSMC0.25, BSMC0.5, BSMC1.0) can be declared to be “high in omega-3 fatty acids”, as they have at least 0.6 g of α-linolenic acid per 100 g of product [45].

#### 3.2.2. Health Indices

Table 6 shows the results obtained for the health indices of the cooked burgers. It can be observed that with the substitution with gelled emulsion (BSM, BSMC0.25, BSMC0.5 and BSMC1.0) in the formulations, the content of omega-3 acids increases with respect to the control (BC), which decreases the ratio of n-6/n-3 (*p* < 0. 05); however, the values are higher in all formulations. This fact may be due to the low content of omega-3 acids in soybean oil, as shown in Table 4 and various studies [19]. However, the omega-6 content increased in the formulations substituted with gelled emulsion, which is beneficial because it replaces saturated fatty acids [6].

The BC formulation showed a PUFA/SFA ratio of 0.19 in the cooked burgers. However, the values of this ratio increased significantly (*p* < 0.05) when substituting the pork backfat with the gelled emulsion. Thus, the BSM formulation showed a value of 0.80, while the samples of BSMC0.25; BSMC0.5, and BSMC1.0 had values ranging between 0.73 and 0.78. This result is considerably beneficial because a PUFA/SFA ratio lower than 0.45 may increase the incidence of cardiovascular diseases [6,46]. The use of gelled emulsion to replace animal fat in several meat products is a great strategy to improve several nutritional indices, including n-6/n-3 and PUFA/SFA ratios, as has been reported by several authors [9,11,41,44]. The partial substitution of pork backfat resulted in a decrease (*p* < 0.05) of the atherogenicity (AI) and thrombogenicity (TI) indices; in all formulations, values less than 1 were found, which indicates that the formulations contribute to reducing the risk and severity of diseases [7]. In addition, higher values of AI and TI (> 1.0) are harmful to human health [47]. The h/H ratio, on the other hand, should be increased. Table 5 shows the formulations with the substitution of pork backfat, which have increased h/H ratios compared to the BC control (*p* < 0.05).

### 3.3. Physico-Chemical Analysis

The physicochemical properties (color, pH, and Aw) of raw and cooked beef burgers are shown in Table 7. Regarding the color properties of raw burgers, the addition of gelled emulsions as fat replacers increased the lightness (L*) and yellowness (b*) values in all samples (*p* < 0.05) with respect to BC. However, no statistical differences were found (*p* > 0.05) between samples where animal fat was replaced by gelled emulsion. In meat products, a higher value of lightness is related to higher free surface water content, which agrees with the moisture values obtained. On the other hand, with respect to BC, the redness (a*) decreased in the raw burgers where the animal fat was substituted with gelled emulsion, although no statistical differences (*p* < 0.0 5) were found. In reference to color differences, the used of gelled emulsion as a fat replacement generates visual changes in color that can be observed by human eyes (ΔE* > 3). These results agree with several studies reporting that the modification or substitution of ingredients as fat can affect color parameters [9,39].

In cooked samples, the use of gelled emulsions had a major impact on redness (a*) and yellowness (b*) coordinates, as well as on the psychophysical parameter C*, since an increase (*p* < 0.05) in the values obtained was observed in all samples substituted with gelled emulsion with respect to BC. Several authors have reported that the use of gelled emulsions in diverse meat products can modify all color parameters. All these differences may be due to the different composition and physicochemical properties of oil, as well as the emulsion characteristics and the rest of ingredients used in the preparation of the meat product [11,39,40,42]. With respect to the differences in instrumental color between the control and the other treatments, it was observed that these increased in the cooked burgers in which the pork backfat was substituted with the gelled emulsion, with ΔE values > 3, meaning that the difference can be perceived by consumers [48].

As can be seen in Table 7, the pH values in the raw burgers ranged from 5.71–5.53; partial substitution decreased the pH compared to the control (*p* < 0.05). On the other hand, due to the heat treatment of cooked burgers, there was an increase in pH, with values ranging between 5.95 and 5.83, and significant differences (*p* < 0.05) between the control and the burgers substituted with gelled emulsion. These results agree with those reported by Lucas-Gonzalez et al. [39]. Furthermore, the substitution of animal fat with several ingredients such as vegetable oils can affect the pH values of reformulated meat products [40,49]. In the case of Aw in raw samples, there were no significant differences (*p* > 0.05) with the substitution of pork backfat with the gelled emulsion. All samples reached intermediate values of food moisture (Aw < 0.90) and several studies show that there are no differences in Aw between raw and cooked burgers [50,51].

### 3.4. Texture Profile and Cooking Properties

**Figure 1:**
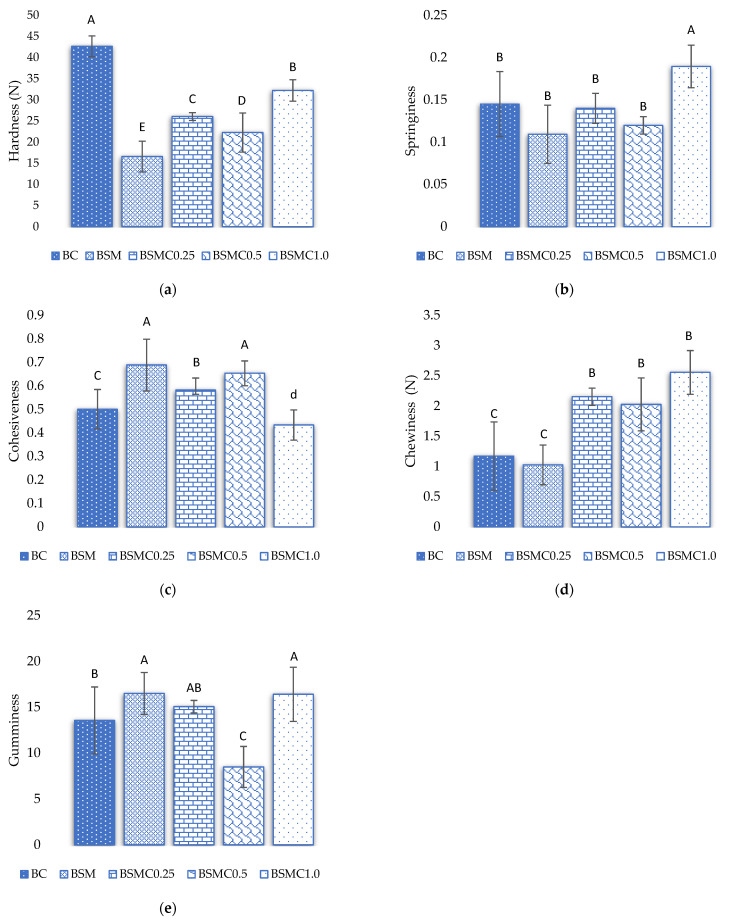
Effect of partial replacement of pork backfat with a gelled emulsion of maca flour, soybean oil, and chincho essential oil on the textural parameters of beef burger. (**a**): hardness; (**b**): Springineness; (**c**): Cohesiveness; (**d**): Chewiness; (**e**): Gumminess. (A-E) Equal capital letters on the same bars indicate that there is no significant different according to Tukey’s HSD post-hoc test (*p* > 0.05). PB: pork backfat; GE: gelled emulsion BC: control hamburger with a traditional formula (20% PB); BSM: burger with 10% PB and 10% substituted by GE_1_ with maca flour and soybean oil; BSMC0.25: burger with 10% PB and 10% substituted by GE_2_ with maca flour, soybean oil, and chincho essential oil; BSMC0.5: burger with 10% PB and 1;% substituted by GE_3_ with maca flour, soybean oil, and chincho essential oil; BSMC1.0: burger with 10% of PB and 10% substituted by GE_4_ with maca flour, soybean oil, and chincho essential oil.

Figure 1 illustrates the influence of various types of gelled emulsion compounds on the texture profile of cooked beef burgers. Regarding the hardness values, significant differences were found in all treatments (*p* < 0.05). The BC formulation presented the highest hardness value; this may be due to the fact that during cooking, the loss of water is related to the generation of hardness, which is apparently influenced by collagen and other myofibrillar proteins [52].

Regarding springiness, no significant differences were found (*p* > 0.05) with the exception of the BSMC1.0 formulation (*p* < 0.05); these data are similar to those reported by Botella-Martinez et al. [9], who found no significant differences when substituting partial pork backfat with a gelled emulsion of amaranth flour with chia or hemp oil. On the contrary, other studies show that there are significant differences in springiness when reformulating burgers with a gelled emulsion [11,41]. The elasticity values may also be due to protein denaturation, which contributes to the higher elasticity in the gels [10]. The BSM formulation partially substituted with maca flour and soybean oil showed the highest value of cohesiveness and gumminess, but the lowest value of chewiness compared to the other formulations. These values agree with those reported by Barros et al. [11], Foggiaro et al. [40], and Heck et al. [42], who reported that cohesiveness and gumminess values increase with the substitution of pork backfat with the gelled emulsion; furthermore, Ref. [11] mentions that these results do not allow direct knowledge of which effect has the greatest influence on the change of texture when reformulating the burgers.

Regarding cooking properties (Table 8), the results obtained indicate that the beef burger formulations substituted with the gelled emulsion had lower cooking and shrinkage loss percentages compared to the control (*p* > 0.05), with no significant differences between the formulations except for the BSMC0.25 formulation (*p* < 0.05). These results are similar to those reported by Heck et al. [42], in which cooking losses were reduced by up to 60% with a gelled emulsion replacing pork backfat (*p* > 0.05); likewise, Refs. [40,49] reported lower cooking losses when reformulating burgers with basil leaf, thyme oil, pistachio oil, and seaweed oil (*p* > 0.05). On the other hand, our results differ from those reported by [9,28], who found significant differences (*p* < 0.05) when reformulating meat burgers with gelled emulsions. In the case of the thickening property, the formulations substituted with the gelled emulsion showed increased thickening compared to the control except for the BSM sample (*p* < 0.05), which presented the lowest values, probably due to its ingredients.

### 3.5. Oxidative Stability

**Figure 2:**
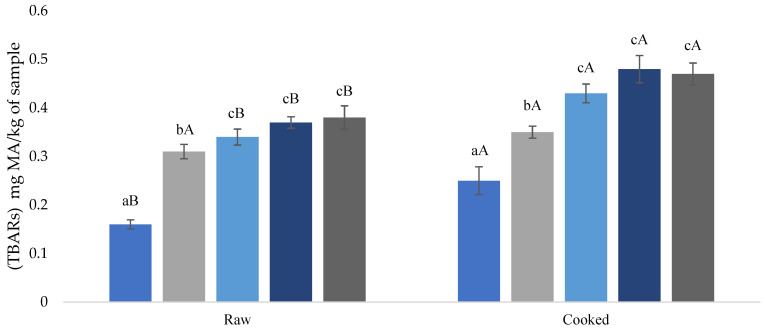
Effect of partial substitution of pork backfat with a gelled emulsion of maca flour, soybean oil, and chincho essential oil on the TBARs values of cooked beef burger. For each thermal treatment (raw or cooked), bars with different small letters indicate the existence of significant differences (*p* < 0.05) among samples (BC, BSM, BSM0.25; BSM0.5, and BSM1.0) according to Tukey’s HSD post-hoc test. For each sample (BC, BSM, BSM0.25; BSM0.5, and BSM1.0), bars with different capital letters indicate the existence of significant differences (*p* < 0.05) among thermal treatments (raw or cooked) according to Tukey’s HSD post-hoc test. PB: pork backfat; GE: gelled emulsion *BC: control burger with a traditional formula (20% PB); BSM: burger with 10% PB and 10% substituted by GE_1_ with maca flour and soybean oil; BSMC0.25: burger with 10% PB and 10% substituted by GE_2_ with maca flour, soybean oil, and chincho essential oil; BSMC0.5: burger with 10% PB and 10% substituted by GE_3_ with maca flour, soybean oil, and chincho essential oil; BSMC1.0: burger with 10% of PB and 10% substituted by GE_4_ with maca flour, soybean oil, and chincho essential oil.

Lipid oxidation is related to spoilage and off-flavors in processed meat products [20,53]. Changes in the TBAR content of partially substituted raw and cooked beef burgers (Figure 2) indicated that TBAR levels were significantly (*p* < 0.05) affected by gelled emulsion substitution. As expected, MDA content increased after cooking (*p* < 0.05), regardless of the type of treatment.

TBAR content had values ranging from 0.16 to 0.38 mg MDA/kg in raw burgers, while in cooked burgers, the range was 0.25 to 0.47 mg MDA/kg. These results agree with those reported by Heck et al. [42], in which TBARs values increase significantly with fat substitution. For their part, Fusaro et al., [21] evaluated oxidative stability in Marchigiana burgers treated with and without a blend of essential oils (*Rosmarinus officinalis* and *Origanum vulgare* var. hirtum). These authors reported that lipid oxidation values were higher (0.55 to 0.43 mg MDA/kg) in burgers without addition and with direct addition of oregano and rosemary essential oil. As expected, the samples where the fat was replaced by gelled emulsions elaborated with soybean oil showed higher lipid oxidation values in both raw and cooked samples compared to the control sample. This fact could be explained by soybean oil being associated with low oxidative stability and rancidity due to its high content of polyunsaturated fatty acids [19].

In all raw and cooked formulations, the acceptability limit (2 mg MDA/kg) for quality loss and lipid oxidation perception by consumers as proposed by Greene and Cumuze [54] was not exceeded, while the addition of chincho essential oil in BSMC0.25, BSMC0.5, and BSMC1.0 formulations showed no significant differences (*p* > 0.05) in the addition of oil concentrations. This may be due to the fact that chincho essential oil presents pro-oxidant activity in concentrations of 0.25–1%; this fact agrees with the results reported by Taherian et al. [55], who mentioned that the use of vegetable oils in emulsions is complex due to the sensitivity to oxidation. An alternative is the addition of various essential oils with high contents of phenolic compounds to reduce lipid oxidation [56]; on the other hand, the reduction of lipid oxidation in formulations substituted with gelled emulsions with the use of carregin as a fat substitute has also been demonstrated [57].

## 4. Conclusions

Replacement of pork backfat with gelled emulsion GE reduced the content of saturated fatty acids (SFA) and increased that of polyunsaturated fatty acids (PUFA) (mainly linoleic acid); in addition, there was a considerable increase in the PUFA/SFA ratio and a decrease of up to 26.53 and 34.45% in the atherogenicity and thrombogenicity indices (BSMC0.5 and BSMC1.0), respectively. The h/H ratio increased to a value of 37% (BSMC1.0).

In addition, the addition of gelled emulsion decreased the amount of fat and protein, and lowered the pH; water activity in raw burgers was not modified. Hardness (*p* < 0.05), cooking losses, shrinkage, and thickness changes decreased with the addition of GE. Lipid oxidation levels were higher in cooked burgers and were significantly affected (*p* < 0.05) by GE substitution.

Therefore, replacing pork backfat with gelled emulsions containing maca flour, soybean oil, and chincho essential oil can be considered as an effective strategy to produce healthier burgers without negatively affecting their physicochemical and technological properties.

## Figures and Tables

**Table 1:** Composition of maca-soybean oil and chincho essential oil gelled emulsions (GE).

Formulations (%)					
Samples *	Water	Instant Gel	Maca Flour	Soy Bean Oil	Chincho Essential Oil
GE_1_	40	5	15	40	0
GE_2_	40	5	15	39.75	0.25
GE_3_	40	5	15	39.5	0.5
GE_4_	40	5	15	39	1

* GE_1_: pork backfat (PB) substituted with maca flour and soybean oil; GE_2_: PB substituted with maca flour, soybean oil, and 0.25% chincho essential oil; GE_3_: PB substituted with maca flour, soybean oil, and 0.5% chincho essential oil; GE_4_: PB substituted with maca flour, soybean oil, and 1.0% of chincho essential oil.

**Table 2:** Formulation of beef burgers with and without gelled emulsions (GE) of maca, soybean oil, and chincho essential oil used as partial substitutes for animal fat.

Treatment * (%)					
	BC	BSM	BSMC0.25	BSMC0.5	BSMC1.0
Beef meat	80	80	80	80	80
Pork backfat	20	10	10	10	10
GE_1_	0	10	0	0	0
GE_2_	0	0	10	0	0
GE_3_	0	0	0	10	0
GE_4_	0	0	0	0	10
Water	5	5	5	5	5
Salt	1.5	1.5	1.5	1.5	1.5
Onion powder	0.3	0.3	0.3	0.3	0.3
Garlic powder	0.3	0.3	0.3	0.3	0.3
Black pepper	0.2	0.2	0.2	0.2	0.2
Dehydrated parsley	0.5	0.5	0.5	0.5	0.5

PB: pork backfat; GE: gelled emulsion. * BC: control burger with a traditional formula (20% pork backfat); BSM: burger with 10% PB and 10% substituted by GE_1_ with maca flour and soybean oil; BSMCC0.25: burger with 10% PB and 10% substituted by GE_2_ with maca flour, soybean oil, and chincho essential oil, BSMC0.5: burger with 10% PB and 10% substituted by GE_3_ with maca flour, soybean oil, and chincho essential oil; BSMC1.0: burger with 10% of PB and 10% substituted by EG_4_ with maca flour, soybean oil, and chincho essential oil.

**Table 3:** Effect of partial substitution of pork backfat by a gelled emulsion of maca flour, soybean oil, and chincho essential oil on the chemical composition (%) of raw and cooked beef burgers.

	Treatments *				
	Raw				
	BC	BSM	BSMC0.25	BSMC0.5	BSMC1.0
Moisture	63.03 ± 0.61 ^a^	65.96 ± 0.39 ^b^	66.01 ± 0.18 ^b^	66.09 ± 0.44 ^b^	65.80 ± 0.40 ^b^
Protein	19.67 ± 0.58 ^b^	18.17 ± 0.00 ^a^	18.26 ± 0.12 ^a^	17.91 ± 0.47 ^a^	18.14 ± 0.09 ^a^
Fat	12.26 ± 0.05 ^c^	7.16 ± 0.15 ^a^	8.36 ± 0.20 ^b^	7.90 ± 0.62 ^a,b^	7.67 ± 0.19 ^a,b^
Ash	2.35 ± 0.07 ^a^	2.46 ± 0.09 ^a^	2.33 ± 0.03 ^a^	2.36 ± 0.07 ^a^	2.25 ± 0.07 ^a^
	**Cooked**				
Moisture	53.69 ± 0.39 ^a^	55.56 ± 0.15 ^b,c^	56.79 ± 0.27 ^c^	55.13 ± 0.84 ^a,b,c^	54.56 ± 1.02 ^a,b^
Protein	27.52 ± 0.03 ^c^	25.16 ± 0.45 ^b^	24.42 ± 0.10 ^a^	24.64 ± 0.07 ^a,b^	25.10 ± 0.16 ^b^
Fat	12.97 ± 0.17 ^b^	12.05 ± 0.27 ^a^	12.18 ± 0.32 ^a,b^	12.76 ± 0.05 ^a,b^	12.06 ± 0.49 ^a^
Ash	2.94 ± 0.05 ^b^	3.00 ± 0.02 ^b^	2.81 ± 0.12 ^a,b^	2.87 ± 0.05 ^a,b^	2.74 ± 0.09 ^a^

^(a–c)^ Equal letters on the same row indicate that there is no significant different according to Tukey’s HSD post-hoc test (*p* > 0.05). PB: pork back fat; GE: gelled emulsion. * BC: control hamburger with a traditional formula (20% PB); BSM: burger with 10% PB and 10% substituted by GE_1_ with maca flour and soybean oil; BSMC0.25: burger with 10% PB and 10% substituted by GE_2_ with maca flour, soybean oil, and chincho essential oil; BSMC0.5: burger with 10% PB and 10% substituted by GE_3_ with maca flour, soybean oil, and chincho essential oil; BSMC1.0: burger with 10% of PB and 10% substituted by GE_4_ with maca flour, soybean oil, and chincho essential oil.

**Table 4:** Effect of partial replacement of pork backfat with a gelled emulsion of maca flour, soybean oil, and chincho essential oil on the fatty acid profile of raw beef burgers.

Fatty Acid (g/100 g of Fat)	Raw				
	Treatment				
	^*^ BC	BSM	BSMC0.25	BSMC0.5	BSMC1.0
C10:0	0.07 ± 0.00 ^c^	0.06 ± 0.00 ^b^	0.05 ± 0.00 ^a,b^	0.05 ± 0.01 ^a^	0.05 ± 0.01 ^a,b^
C12:0	0.08 ± 0.00 ^c^	0.06 ± 0.00 ^b^	0.06 ± 0.00 ^a,b^	0.06 ± 0.0 ^a^	0.06 ± 0.00 ^a,b^
C14:0	1.5 ± 0.04 ^b^	1.17 ± 0.09 ^a^	1.13 ± 0.03 ^a^	1.14 ± 0.11 ^a^	1.14 ± 0.01 ^a^
C14:1 (n-5)	0.04 ± 0.03 ^a^	0.05 ± 0.02 ^a^	0.10 ± 0.12 ^a^	0.08 ± 0.11 ^a^	0.06 ± 0.01 ^a^
C15:0	0.08 ± 0.01 ^a^	0.06 ± 0.03 ^a^	0.08 ± 0.00 ^a^	0.09 ± 0.01 ^a^	0.08 ± 0.00 ^a^
C16:0	23.88 ± 0.00 ^b^	20.52 ± 0.48 ^a^	19.95 ± 0.27 ^a^	19.86 ± 0.80 ^a^	19.93 ± 0.12 ^a^
C16:1 (n-7)	2.38 ± 0.06 ^b^	1.88 ± 0.10 ^a^	1.76 ± 0.03 ^a^	1.75 ± 0.11 ^a^	1.80 ± 0.02 ^a^
C17:0	0.42 ± 0.02 ^b^	0.34 ± 0.01 ^a^	0.34 ± 0.00 ^a^	0.36 ± 0.03 ^a^	0.35 ± 0.01 ^a^
C17:1 (n-7)	0.41 ± 0.00 ^c^	0.32 ± 0.01 ^a,b^	0.31 ± 0.01 ^a^	0.33 ± 0.02 ^a,b^	0.33 ± 0.01 ^b^
C18:0	11.42 ± 0.13 ^b^	9.49 ± 0.36 ^a^	9.13 ± 0.24 ^a^	9.31 ± 0.52 ^a^	9.20 ± 0.19 ^a^
C18:1 (n-9)Cis	48.68 ± 0.20 ^d^	43.12 ± 0.01 ^c^	41.80 ± 0.16 ^a^	41.42 ± 0.58 ^a^	42.46 ± 0.41 ^b^
C18:1 (n-9)Trans	2.58 ± 0.07 ^d^	2.29 ± 0.03 ^c^	2.21 ± 0.02 ^a,b^	2.15 ± 0.02 ^a^	2.25 ± 0.03 ^b,c^
C18:2 (n-6)	6.20 ± 0.10 ^a^	16.95 ± 0.89 ^b^	19.63 ± 0.29 ^c^	19.96 ± 1.85 ^c^	18.61 ± 0.09 ^b,c^
C18:3 (n-3)	0.32 ± 0.00 ^a^	1.60 ± 0.07 ^b^	1.92 ± 0.01 ^c^	1.95 ± 0.19 ^c^	1.79 ± 0.01 ^c^
C18:3 (n-6)	0.17 ± 0.00 ^a^	0.23 ± 0.00 ^b^	0.24 ± 0.01 ^c^	0.25 ± 0.01 ^c^	0.24 ± 0.00 ^c^
C20:0	1.19 ± 0.01 ^d^	0.90 ± 0.02 ^c^	0.80 ± 0.02 ^a^	0.79 ± 0.00 ^a^	0.86 ± 0.02 ^b^
C20:1	0.34 ± 0.00 ^b^	0.23 ± 0.02 ^a^	0.21 ± 0.03 ^a^	0.22 ± 0.00 ^a^	0.24 ± 0.02 ^a^
C20:3 (n-8)	0.15 ± 0.12 ^a^	0.34 ± 0.03 ^b^	0.18 ± 0.03 ^a^	0.18 ± 0.10 ^a^	0.37 ± 0.04 ^b^
C20:3 (n-11)	0.04 ± 0.03 ^a,b^	0.18 ± 0.08 ^c^	0.04 ± 0.01 ^a,b^	0.02 ± 0.02 ^a^	0.14 ± 0.09 ^b,c^
C24:1	0.05 ± 0.05 ^a^	0.18 ± 0.03 ^b^	0.09 ± 0.09 ^a,b^	0.04 ± 0.04 ^a^	0.05 ± 0.04 ^a^
ΣSFA	38.65 ± 0.19 ^b^	32.60 ± 0.95 ^a^	31.53 ± 0.51 ^a^	31.65 ± 1.48 ^a^	31.67 ± 0.30 ^a^
ΣMUFA	54.42 ± 0.29 ^d^	47.89 ± 0.12 ^c^	46.40 ± 0.03 ^a,b^	45.95 ± 0.73 ^a^	47.14 ± 0.48 ^b,c^
ΣPUFA	6.88 ± 0.05 ^a^	19.31 ± 1.01 ^b^	22.01 ± 0.34 ^c^	22.36 ± 2.17 ^c^	21.14 ± 0.22 ^b,c^
Σn-3	0.32 ± 0.00 ^s^	1.60 ± 0.07 ^b^	1.92 ± 0.01 ^c^	1.95 ± 0.19 ^c^	1.79 ± 0.01 ^c^
Σn-6	6.37 ± 0.10 ^a^	17.18 ± 0.89 ^b^	19.88 ± 0.29 ^c^	20.21 ± 1.86 ^c^	18.85 ± 0.10 ^b,c^

Results are expressed as g/100 g. Data are presented as mean ± standard deviation. ^(a–d)^ For each parameter, results followed by the same letter are not significantly different according to Tukey’s HSD post-hoc test (*p* > 0.05). A lower-case letters refers to the comparison of the same fatty acid or parameters between the different raw samples. PB: pork backfat; GE: gelled emulsion.* BC: control hamburger with a traditional formula (20% PB); BSM: burger with 10% PB and 10% substituted by GE_1_ with maca flour and soybean oil; BSMC0.25: burger with 10% PB and 10% substituted by GE_2_ with maca flour, soybean oil, and chincho essential oil; BSMC0.5: burger with 10% PB and 10% substituted by GE_3_ with maca flour, soybean oil, and chincho essential oil; BSMC1.0: burger with 10% of PB and 10% substituted by GE_4_ with maca flour, soybean oil, and chincho essential oil. SFA = saturated fatty acids; MUFA = monounsaturated fatty acids; PUFA = polyunsaturated fatty acids; n-6 = omega-6; n-3 = omega-3.

**Table 5:** Effect of partial replacement of pork backfat with a gelled emulsion of maca flour, soybean oil, and chincho essential oil on the fatty acid profile of cooked beef burgers.

Fatty Acid (g/100 g of Fat)	Cooked				
	Treatment *				
	BC	BSM	BSMC0.25	BSMC0.5	BSMC1.0
C10:0	0.08 ± 0.01 ^b^	0.05 ± 0.00 ^a^	0.05 ± 0.00 ^a^	0.05 ± 0.00 ^a^	0.05 ± 0.00 ^a^
C12:0	0.08 ± 0.00 ^b^	0.05 ± 0.00 ^a^	0.05 ± 0.00 ^a^	0.05 ± 0.00 ^a^	0.06 ± 0.00 ^a^
C14:0	1.48 ± 0.02 ^d^	1.01 ± 0.01 ^a^	1.07 ± 0.00 ^b^	1.12 ± 0.00 ^c^	1.13 ± 0.02 ^c^
C14:1 (n-5)	0.06 ± 0.01 ^a^	0.08 ± 0.02 ^a^	0.08 ± 0.00 ^a^	0.08 ± 0.01 ^a^	0.08 ± 0.01 ^a^
C15:0	0.09 ± 0.00 ^b^	0.04 ± 0.04 ^a^	0.09 ± 0.00 ^b^	0.09 ± 0.00 ^b^	0.09 ± 0.00 ^b^
C16:0	23.68 ± 0.03 ^c^	19.20 ± 0.10 ^a^	19.42 ± 0.03 ^a^	19.70 ± 0.13 ^b^	19.70 ± 0.24 ^b^
C16:1 (n-7)	2.41 ± 0.02 ^c^	1.68 ± 0.00 ^a^	1.69 ± 0.00 ^a^	1.75 ± 0.03 ^b^	1.76 ± 0.03 ^b^
C17:0	0.43 ± 0.00 ^d^	0.33 ± 0.00 ^a^	0.34 ± 0.00 ^b^	0.36 ± 0.01 ^c^	0.36 ± 0.00 ^c^
C17:1 (n-7)	0.42 ± 0.00 ^d^	0.31 ± 0.00 ^b^	0.30 ± 0.00 ^a^	0.33 ± 0.00 ^c^	0.33 ± 0.00 ^c^
C18:0	11.86 ± 0.08 ^c^	9.03 ± 0.02 ^a^	9.25 ± 0.03 ^a,b^	9.36 ± 0.13 ^b^	9.35 ± 0.23 ^b^
C18:1 (n-9)Cis	47.92 ± 0.02 ^b^	40.70 ± 0.15 ^a^	40.44 ± 0.02 ^a^	41.00 ± 0.50 ^a^	40.97 ± 0.46 ^a^
C18:1 (n-9)Trans	2.57 ± 0.08 ^b^	2.18 ± 0.03 ^a^	2.14 ± 0.02 ^a^	2.17 ± 0.01 ^a^	2.13 ± 0.06 ^a^
C18:2 (n-6)	6.46 ± 0.04 ^a^	21.50 ± 0.06 ^c^	21.48 ± 0.15 ^c^	20.37 ± 0.75 ^b^	20.33 ± 0.88 ^b^
C18:3 (n-3)	0.30 ± 0.01 ^a^	2.05 ± 0.02 ^c^	2.06 ± 0.02 ^c^	1.91 ± 0.07 ^b^	1.92 ± 0.11 ^b^
C18:3 (n-6)	0.17 ± 0.00 ^a^	0.26 ± 0.00 ^c^	0.26 ± 0.00 ^c^	0.24 ± 0.01 ^b^	0.25 ± 0.00 ^b^
C20:0	1.14 ± 0.02 ^c^	0.76 ± 0.00 ^b^	0.71 ± 0.00 ^a^	0.75 ± 0.03 ^b^	0.76 ± 0.02 ^b^
C20:1	0.33 ± 0.01 ^b^	0.21 ± 0.05 ^a,b^	0.14 ± 0.15 ^a^	0.14 ± 0.11 ^a^	0.20 ± 0.01 ^a,b^
C20:3 (n-8)	0.37 ± 0.01 ^a^	0.42 ± 0.00 ^b^	0.36 ± 0.01 ^a^	0.44 ± 0.01 ^b^	0.45 ± 0.03 ^b^
C20:3 (n-11)	0.08 ± 0.04 ^b^	0.03 ± 0.00 ^a^	0.02 ± 0.00 ^a^	0.03 ± 0.01 ^a^	0.02 ± 0.00 ^a^
C24:1	0.06 ± 0.02 ^a^	0.11 ± 0.10 ^a^	0.07 ± 0.01 ^a^	0.07 ± 0.07 ^a^	0.07 ± 0.07 ^a^
ΣSFA	38.84 ± 0.04 ^d^	30.48 ± 0.08 ^a^	30.97 ± 0.00 ^b^	31.48 ± 0.27 ^c^	31.49 ± 0.47 ^c^
ΣMUFA	53.71 ± 0.04 ^b^	45.15 ± 0.10 ^a^	44.78 ± 0.18 ^a^	45.46 ± 0.63 ^a^	45.47 ± 0.55 ^a^
ΣPUFA	7.39 ± 0.10 ^a^	24.26 ± 0.08 ^c^	24.17 ± 0.17 ^c^	22.99 ± 0.83 ^b^	22.97 ± 0.96 ^b^
Σn-3	0.30 ± 0.01 ^a^	2.05 ± 0.02 ^c^	2.06 ± 0.02 ^c^	1.91 ± 0.07 ^b^	1.92 ± 0.11 ^b^
Σn-6	6.63 ± 0.04 ^a^	21.76 ± 0.06 ^c^	21.73 ± 0.15 ^c^	20.61 ± 0.75 ^b^	20.58 ± 0.88 ^b^

Results are expressed as g/100 g. Data are presented as mean ± standard deviation. ^(a–d)^ For each parameter, results followed by same letter are not significantly different according to Tukey’s HSD post-hoc test (*p* > 0.05). A lower-case letters refers to the comparison of the same fatty acid or parameters between the different cooked samples. PB: pork backfat; GE: gelled emulsion * BC: control hamburger with a traditional formula (20% PB); BSM: burger with 10% PB and 10% substituted by GE_1_ with maca flour and soybean oil; BSMC0.25: burger with 10% PB and 10% substituted by GE_2_ with maca flour, soybean oil, and chincho essential oil; BSMC0.5: burger with 10% PB and 10% substituted by G_E3_ with maca flour, soybean oil, and chincho essential oil; BSMC1.0: burger with 10% of PB and 10% substituted by GE_4_ with maca flour, soybean oil, and chincho essential oil. SFA = saturated fatty acids; MUFA = monounsaturated fatty acids; PUFA = polyunsaturated fatty acids; n-6 = omega-6; n-3 = omega-3.

**Table 6:** Health indices of cooked beef burgers reformulated with gelled emulsion of maca flour, soybean oil, and chincho essential oil used as substitutes for pork backfat.

Indices	Formulation *				
	BC	BSM	BSMC0.25	BSMC0.5	BSMC1.0
n-6/n-3	21.83 ± 0.32 ^b^	10.63 ± 0.06 ^a^	10.53 ± 0.01 ^a^	10.79 ± 0.01 ^a^	10.73 ± 0.15 ^a^
PUFA/SFA	0.19 ± 0.00 ^a^	0.80 ± 0.00 ^c^	0.78 ± 0.01 ^c^	0.73 ± 0.03 ^b^	0.73 ± 0.04 ^b^
AI	0.49 ± 0.00 ^d^	0.34 ± 0.00 ^a^	0.35 ± 0.00 ^b^	0.36 ± 0.00 ^c^	0.36 ± 0.00 ^c^
TI	1.19 ± 0.00 ^c^	0.74 ± 0.00 ^a^	0.75 ± 0.00 ^a^	0.78 ± 0.01 ^b^	0.78 ± 0.02 ^b^
h/H	2,29 ± 0.01 ^b^	2.98 ± 0.12 ^a^	3.13 ± 0.06 ^a^	3.14 ± 0.21 ^a^	3.12 ± 0.03 ^a^

^(a–c)^ Equal letters on the same row indicate that there is no significant different according to Tukey’s HSD post-hoc test (*p* > 0.05). PB: pork back fat; GE: gelled emulsion. * BC: control hamburger with a traditional formula (20% PB); BSM: burger with 10% PB and 10% substituted by GE1 with maca flour and soybean oil; BSMC0.25: burger with 10% PB and 10% substituted by GE2 with maca flour, soybean oil, and chincho essential oil; BSMC0.5: burger with 10% PB and 10% substituted by GE3 with maca flour, soybean oil, and chincho essential oil; BSMC1.0: burger with 10% of PB and 10% substituted by GE4 with maca flour, soybean oil, and chincho essential oil. SFA: saturated fatty acids. PUFA: polyunsaturated fatty acids. n-3: omega-3; n-6: omega-6; AI: atherogenic index; TI: thrombogenic index; h/H: hypo cholesterolemic/hypercholesterolemic index.

**Table 7:** Effect of partial substitution of pork backfat by a gelled emulsion of maca flour, soybean oil, and chincho essential oil on the color, pH, and Aw parameters of raw and cooked beef burger.

	Treatments *				
			Raw		
	BC	BSM	BSMC0.25	BSMC0.5	BSMC1.0
L*	44.35 ± 0.99 ^a^	48.52 ± 2.11 ^b^	49.28 ± 1.89 ^b^	49.82 ± 2.64 ^b^	48.22 ± 2.32 ^b^
a*	7.62 ± 1.33 ^a^	6.82 ± 1.12 ^a^	6.21 ± 0.68 ^a^	6.20 ± 1.24 ^a^	6.56 ± 1.28 ^a^
b*	13.31 ± 1.44 ^a^	14.18 ± 0.75 ^a,b^	15.58 ± 1.09 ^c^	14.82 ± 1.14 ^b,c^	15.34 ± 1.17 ^b,c^
C*	15.41 ± 1.36 ^a^	15.77 ± 0.90 ^a,b^	16.79 ± 1.06 ^b^	16.11 ± 1.17 ^a,b^	16.74 ± 0.97 ^b^
H*	60.13 ± 5.34 ^a^	64.37 ± 3.64 ^a,b^	68.22 ± 2.48 ^b^	67.32 ± 4.35 ^b^	66.79 ± 4.90 ^b^
ΔE*	-	4.98 ± 2.10 ^a^	6.09 ± 1.18 ^a^	6.68 ± 3.10 ^a^	5.18 ± 1.97 ^a^
pH	5.71 ± 0.01 ^c^	5.68 ± 0.01 ^c^	5.62 ± 0.01 ^b^	5.53 ± 0.05 ^a^	5.62 ± 0.00 ^b^
aw	0.89 ± 0.0 ^a^	0.89 ± 0.0 ^a^	0.89 ± 0.0 ^a^	0.89 ± 0.0 ^a^	0.89 ± 0.0 ^a^
	**Cooked**				
	**BC**	**BSM**	**BSMC0.25**	**BSMC0.5**	**BSMC1.0**
L*	41.46 ± 5.04 ^a^	43.12 ± 6.00 ^a^	40.74 ± 4.59 ^a^	42.41 ± 4.44 ^a^	41.54 ± 4.08 ^a^
a*	4.32 ± 1.28 ^a^	5.60 ± 1.21 ^b^	6.31 ± 1.13 ^b^	5.61 ± 1.14 ^b^	6.51 ± 0.96 ^b^
b*	8.32 ± 3.82 ^a^	12.62 ± 2.15 ^b^	12.6 ± 2.13 ^b^	11.71 ± 2.54 ^b^	13.02 ± 1.97 ^b^
C*	9.73 ± 3.03 ^a^	13.93 ± 1.59 ^b^	14.20 ± 1.73 ^b^	13.12 ± 2.08 ^b^	14.63 ± 1.65 ^b^
H	60.12 ± 15.43 ^a^	65.36 ± 8.52 ^a^	62.86 ± 6.91 ^a^	63.42 ± 8.70 ^a^	63.03 ± 5.69 ^a^
ΔE*	-	8.40 ± 5.50 ^a^	8.57 ± 4.54 ^a^	7.62 ± 3.28 ^a^	8.03 ± 4.20 ^a^
pH	5.95 ± 0.03 ^b^	5.87 ± 0.02 ^a^	5.83 ± 0.03 ^a^	5.83 ± 0.02 ^a^	5.86 ± 0.04 ^a^
aw	-	-	-	-	-

^(a–c)^ Equal letters on the same row indicate that there is no significant different according to Tukey’s HSD post-hoc test (*p* > 0.05). PB: pork backfat; GE: gelled emulsion. - non determined; * BC: control hamburger with a traditional formula (20% PB); BSM: burger with 10% PB and 10% substituted by GE_1_ with maca flour and soybean oil; BSMC0.25: burger with 10% PB and 10% substituted by GE_2_ with maca flour, soybean oil, and chincho essential oil; BSMC0.5: burger with 10% PB and 10% substituted by GE_3_ with maca flour, soybean oil, and chincho essential oil; BSMC1.0: burger with 10% of PB and 10% substituted by GE_4_ with maca flour, soybean oil, and chincho essential oil.

**Table 8:** Effect of partial replacement of pork backfat with a gelled emulsion of maca flour, soybean oil, and chincho essential oil on the cooking characteristics of beef burgers.

			Treatments *		
Technological Parameters (%)	BC	BSM	BSMC0.25	BSMC0.5	BSMC1.0
Cooking loss	28.97 ± 2.5 ^b^	26.47 ± 0.63 ^a,b^	23.39 ± 2.22 ^a^	27.05 ± 1.41 ^a,b^	27.46 ± 0.84 ^b^
Shrinkage	24.68 ± 2.62 ^b^	20.63 ± 2.64 ^a,b^	18.64 ± 1.35 ^a^	19.84 ± 3.09 ^a,b^	22.09 ± 3.52 ^b^
Thickness increase	30.30 ± 3.09 ^b,c^	15.96 ± 2.37 ^a^	48.14 ± 3.20 ^d^	42.81 ± 2.31 ^c,d^	31.66 ± 2.35 ^b^

^(a–d)^ Equal letters on the same row indicate that there is no significant different according to Tukey’s HSD post-hoc test (*p* > 0.05). PB: pork backfat; GE: gelled emulsion.* BC: control hamburger with a traditional formula (20% PB); BSM: burger with 10% PB and 10% substituted by GE_1_ with maca flour and soybean oil; BSMC0.25: burger with 10% PB and 10% substituted by GE_2_ with maca flour, soybean oil, and chincho essential oil; BSMC0.5: burger with 10% PB and 10% substituted by GE_3_ with maca flour, soybean oil, and chincho essential oil; BSMC1.0: burger with 10% of PB and 10% substituted by GE_4_ with maca flour, soybean oil, and chincho essential oil.

## Data Availability

The data presented in this study are available on request from the corresponding author.
